# Mice Harboring a Non-Functional *CILK1/ICK* Allele Fail to Model the Epileptic Phenotype in Patients Carrying Variant *CILK1/ICK*

**DOI:** 10.3390/ijms22168875

**Published:** 2021-08-18

**Authors:** Kathryn A. Salvati, Ashley J. Mason, Casey D. Gailey, Eric J. Wang, Zheng Fu, Mark P. Beenhakker

**Affiliations:** 1 Department of Pharmacology, University of Virginia, Charlottesville, VA 22908, USA; kas5dv@virginia.edu (K.A.S.); ajm4vd@virginia.edu (A.J.M.); cdg7sf@virginia.edu (C.D.G.); ew8sk@virginia.edu (E.J.W.); 2 Department of Neurological Surgery and Weill Institute for Neuroscience, University of California, San Francisco, CA 94143, USA; 3 UVA Cancer Center, Cancer Biology Program, University of Virginia, Charlottesville, VA 22908, USA; 4 UVA Brain Institute, University of Virginia, Charlottesville, VA 22908, USA

**Keywords:** epilepsy, ciliopathy, primary cilium, kinase, seizure, opisthotonos

## Abstract

CILK1 (ciliogenesis associated kinase 1)/ICK (intestinal cell kinase) is a highly conserved protein kinase that regulates primary cilia structure and function. *CILK1* mutations cause a wide spectrum of human diseases collectively called ciliopathies. While several *CILK1* heterozygous variants have been recently linked to juvenile myoclonic epilepsy (JME), it remains unclear whether these mutations cause seizures. Herein, we investigated whether mice harboring either a heterozygous null *Cilk1* (*Cilk1*^+/−^) mutation or a heterozygous loss-of-function *Cilk1* mutation (*Cilk1*^R272Q/+^) have epilepsy. We first evaluated the spontaneous seizure phenotype of *Cilk1*^+/−^ and *Cilk1*^R272Q/+^ mice relative to wildtype littermates. We observed no electrographic differences among the three mouse genotypes during prolonged recordings. We also evaluated electrographic and behavioral responses of mice recovering from isoflurane anesthesia, an approach recently used to measure seizure-like activity. Again, we observed no electrographic or behavioral differences in control versus *Cilk1*^+/−^ and *Cilk1*^R272Q/+^ mice upon isoflurane recovery. These results indicate that mice bearing a non-functional copy of *Cilk1* fail to produce electrographic patterns resembling those of JME patients with a variant *CILK1* copy. Our findings argue against *CILK1* haploinsufficiency being the mechanism that links *CILK1* variants to JME.

## 1. Introduction

The primary cilium is a single solitary microtubule-based protrusion on the apical surface of most vertebrate cells that senses and transduces environmental and hormonal signals to regulate diverse cellular processes [1]. The primary cilium is essential for tissue development and homeostasis. Defects in cilia have been linked to at least 35 human diseases collectively called ciliopathies that manifest as a constellation of clinical features and deformities in various organ systems, including the brain [2]. 

Ciliogenesis-associated kinase 1 (CILK1), formerly known as intestinal cell kinase (ICK), is a highly conserved serine/threonine kinase that negatively regulates cilia length and ciliogenesis [3]. Inactivating mutations in the human *CILK1* gene (R272Q) cause neonatal–lethal human ciliopathies, such as endocrine-cerebro-osteodysplasia (ECO) syndrome [4] and ECO-like syndromes [5,6]. Similar to humans with ECO syndrome, homozygous *Cilk1* null mice and R272Q mutant mice are perinatal lethal [7,8,9]. Complete *Cilk1* deletion also recapitulates ciliopathy phenotypes and causes exuberant cilia growth, consistent with the role of CILK1 in restricting cilia formation and growth. In contrast, cells from heterozygous *Cilk1* null mice grow normal cilia and the mice develop normally [7,8], thus indicating that at least one wildtype copy of the gene is sufficient for normal cilia growth. Collectively, these results support the general conclusion that CILK1 mutations causing exuberant cilia growth result in ciliopathy, whereas CILK1 mutations preserving normal cilia growth are associated with normal development.

Although ample evidence demonstrates that primary cilia critically regulate the development and patterning of the nervous system, cilia function in mature neurons remains poorly understood and underexplored [10]. Moreover, the role of CILK1 in regulating cilia growth and cellular function in postmitotic differentiated cells, such as neurons, remains unknown. Recently, an expansive genetic study linked multiple pathological *CILK1* point mutations to juvenile myoclonic epilepsy (JME) [11], the most commonly diagnosed *Genetic Generalized Epilepsy*. The authors examined the genomes of 334 families whose members are afflicted with JME and identified 21 pathogenic *CILK1* variants in 22 of 310 JME patients. Notably, four *CILK1* variants, K220E, K305T, A615T, and R632X, were strongly linked to JME. Mouse neocortices transfected with these four pathogenic variants and the *CILK1* variant, R272Q, exhibited impaired mitosis, cell-cycle exit, radial neuroblast migration, and increased apoptosis [11]. The authors also evaluated the spontaneous and isoflurane-provoked seizure phenotype of heterozygous *Cilk1* null (*Cilk1*^+/−^) mice and concluded that the mutations are epileptogenic. These novel observations, however, diverge from previous reports on the lack of cilia and development phenotypes in the heterozygous *Cilk1* null mice. 

In this study, we re-examine the hypothesis that CILK1 mutations contribute to JME. We evaluate the seizure phenotype of both heterozygous null *Cilk1* (*Cilk1*^+/−^) mice and mice harboring the loss-of-function *Cilk1* variant, R272Q (*Cilk1*^R272Q/+^) [9,12]. By coupling video capture with electrocorticogram and electromyogram (ECoG/EMG) recordings, we first evaluated the spontaneous seizure phenotype of both Cilk1 mutant mice. We captured no electrographic or behavioral tonic–clonic seizures in *Cilk1*^+/−^ and *Cilk1*^R272Q/+^ mice. Second, we investigated whether isoflurane exposure induces tonic–clonic seizures in *Cilk1*^+/−^ and *Cilk1*^R272Q/+^ mice, as previously reported [11]. Again, we observed no seizures during the recovery phase of isoflurane treatment. However, a subset of mice exhibited opisthotonos-like behavior during isoflurane recovery, a nonepileptic behavior that includes tonic full-body extension but is electrographically normal. In sum, our observations do not support the hypothesis that either *Cilk1*^+/−^ or *Cilk1*^R272Q/+^ mutations promote JME. These results challenge the recent conclusion that *CILK1* haploinsufficiency contributes to JME. 

## 2. Results

### 2.1. Cilk1^+/−^ and Cilk1^R272Q/+^ Mice do Not Exhibit Electrographic Seizures

**Figure 1:**
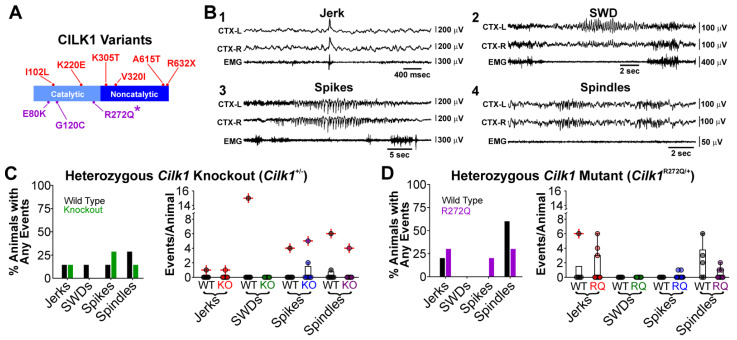
The seizure phenotype of *Cilk1*^+/−^ and *Cilk1*^R272Q/+^ mutants is indistinguishable from wildtype littermates. (**A**) Cilk1 mutations associated with JME (red) and those associated with ciliopathies (purple). Asterisk indicates the *CILK1* mutation (R272Q) that our mouse model in (D) carries. (**B**) Electrographic examples of seizure activity observed in mice: (1) myoclonic jerk, (2) spike-wave discharge (SWD), (3) spike run (associated with convulsions), and (4) sleep spindles. (**C**) *Left*. The percentage of *Cilk1*^+/−^ and wildtype littermates that exhibited at least one electrographic jerk, SWD, spike run, or spindle. *Right.* The number of electrographic events per animal. (**D**) *Left*. The percentage of *Cilk1*^R272Q/+^ and wildtype littermates that exhibited at least one electrographic jerk, SWD, spike run, or spindle. *Right.* The number of electrographic events per animal. Symbols marked with a red cross are considered to be outliers according to Matlab-based functions.

CILK1 has two structural domains, a catalytic N-terminal domain (amino acids 4-284) and a noncatalytic C-terminal domain (amino acids 285–632). *CILK1* variants associate with both JME [11] (Figure 1A, red) and human ciliopathies [4,5,6] (Figure 1A, purple). Notably, recent evidence calls into question the association between *CILK1* variants and JME [13]. Moreover, previous reports indicate that *Cilk1* heterozygote knockout (i.e., *Cilk1*^+/−^) mice develop normally and exhibit normal cilia growth [7,8]. We were therefore motivated to re-evaluate the hypothesis that *Cilk1*^+/−^ mice generate seizures associated with JME. We compared the seizure phenotype of *Cilk1*^+/−^ mice to wildtype littermates as well as mice harboring the autosomal-recessive inactivating mutation *Cilk1* R272Q (i.e., heterozygous *Cilk1*^R272Q/+^) associated with human ECO syndrome [9]. 

We first evaluated the spontaneous seizure phenotype of *Cilk1*^+/−^ and *Cilk1*^R272Q/+^ mutant mice with their respective wildtype littermates using chronic video-ECoG/EMG recordings. Common electrographic events observed in JME patients include myoclonic jerks, generalized tonic–clonic seizures, polyspikes, and absence seizures. These events have readily observable correlates in mice (Figure 1B). We also catalogued sleep spindles (Figure 1B) to determine if Cilk1 mutations contribute to abnormal sleep activity among the three mouse types (i.e., *Cilk1*^+/−^, *Cilk1*^R272Q/+^, and wildtype). Investigators were blinded to the genotype of mice throughout the experimental procedure and data analysis.

By quantifying a 48 h recording period, we concluded that a minority of *Cilk1*^+/−^ and wildtype mice express a low frequency of JME-associated electrographic events. Between 14% (*n* = 1/7) and 29% (*n* = 2/7) of *Cilk1*^+/−^ mice produced myoclonic jerks and spikes, respectively, but no SWDs (*n* = 0/7). Similarly, 14% (1/7) of wildtype littermates exhibited myoclonic jerks and spikes; one mouse had an SWD (*n* = 1/7; Figure 1C, left). We also observed sleep spindles in 14% (*n* = 1/7) of *Cilk1*^+/−^ and 29% (*n* = 2/7) of wildtype mice (Figure 1C, left). Notably, sleep spindles are generally difficult to resolve in rodents and often require automated algorithms for detection [14]; as the primary goal of this study was to evaluate seizures, we did not utilize such spindle detection algorithms. Statistical comparisons revealed that the occurrence of all seizure subtypes did not differ between *Cilk1*^+/−^ and wildtype littermates (myoclonic jerks, *p* = 1.0; SWDs, *p* = 0.37; spikes, *p* = 0.65, spindles, *p* = 0.68; see Figure 1C, right). Similarly, *Cilk1*^R272Q/+^ and their wildtype littermates had minimal JME-associated electrographic activity. Between 30% (*n* = 2/10) and 20% (*n* = 3/10) of *Cilk1*^R272Q/+^ mice had myoclonic jerks and spikes, respectively, and no SWDs (*n* = 0/7; Figure 1D, left). Comparatively, 20% (*n* = 1/5) and 60% (*n* = 3/5) of wildtype littermates exhibited myoclonic jerks and spikes, respectively, and no SWDs (*n* = 0/7; Figure 1D, left). Sleep spindles were observed in 29% (*n* = 2/7) of *Cilk1*^R272Q/+^ and 57% (*n* = 4/7) of wildtype mice (Figure 1D, right). Statistical measures revealed no difference in seizure phenotype between the *Cilk1*^R272Q/+^ and wildtype littermates (myoclonic jerks, *p* = 0.94; SWDs, *p* = 1; spikes, *p* = 0.32). Sleep spindle occurrence was lower in *Cilk1*^R272Q/+^ mice relative to their wildtype littermates (spindles, *p* = 0.047; see Figure 1D, right). Importantly, we did not observe electrographic or behavioral generalized tonic–clonic seizures in any *Cilk1*^+/−^ and *Cilk1*^R272Q/+^ mice, or their wildtype littermates. We also did not observe such seizures while handling or caring for mice. In sum, our data suggest that *Cilk1*^+/−^ and *Cilk1*^R272Q/+^ mice do not have a higher occurrence in JME-like events relative to wildtype mice.

### 2.2. Isoflurane Does Not Induce Generalized Tonic–Clonic Seizures in Cilk1^+/−^ and Cilk1^R272Q/+^ Mice

Isoflurane is a widely used volatile anesthetic for the induction and maintenance of general anesthesia in humans [15,16] and research animals [17]. Generally, isoflurane does not induce seizures in humans and, in fact, exhibits anticonvulsant properties. However, several studies report that emergence from isoflurane anesthesia in mice causes opisthotonos [18,19,20], a behavioral repertoire that includes hyperextension of the neck, arching of the back, and tail extension. Importantly, opisthotonos is not considered to reflect epilepsy [21]. Indeed, opisthotonos in humans is often associated with psychogenic nonepileptic seizures [21,22]. Recently, *Cilk1*^+/−^ mice were reported to generate tonic–clonic seizures upon recovery from isoflurane [11], an assessment based entirely on behavioral measures. As tonic–clonic seizures and opisthotonos include similar behavioral features, we were motivated to test the hypothesis that *Cilk1*^+/−^ mice generate opisthotonos, not seizures, upon isoflurane recovery, a distinction readily testable using ECoG/EMG recordings.

**Figure 2:**
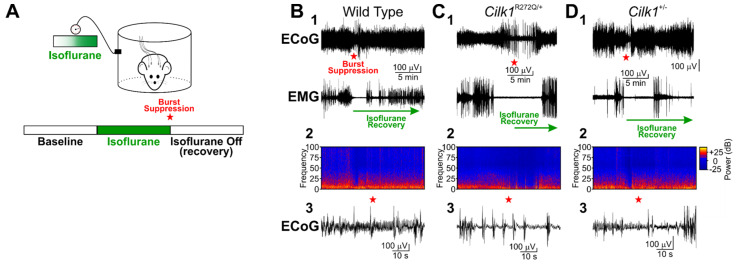
ECoG/EMG recordings in *Cilk1*^+/−^ and *Cilk1*^R272Q/+^ mutants during and after isoflurane exposure is consistent with opisthotonos, not JME. (**A**) Mice were placed in a plethysmography chamber that enabled controlled delivery of isoflurane. Baseline ECoG/EMG activity was recorded for 45 min, after which isoflurane (1.5% or 5%) was delivered to the chamber. Once burst suppression was observed in the ECoG, isoflurane delivery was halted and the animal was allowed to recover. Opisthotonos generally occurs within a few minutes of isoflurane recovery. (**B**–**D**) ECoG/EMG activity of a (**B**) wildtype, (**C**) *Cilk1*^+/−,^ and (**D**) *Cilk1*^R272Q/+^ mouse before, during, and after 1.5% isoflurane exposure. (1) Compressed recordings of ECoG/EMG. Red star indicates occurrence of burst suppression. Green arrow indicates period of isoflurane recovery. Note the suppression of all muscle activity surrounding burst suppression occurrence. (2) Spectrogram of ECoG recording in (1). ECoG signals are generally enriched in low-frequency components indicative of resting or sleeping behaviors. The spectrograms show no generalized tonic–clonic activity. (3) Expanded ECoG trace showing burst suppression.

To record ECoG/EMG activity in behaving mice before and during isoflurane exposure, we used plethysmography chambers to reduce the variability of isoflurane gas exchange among mice. While recording ECoG/EMG signals in *Cilk1*^+/−^ and *Cilk1*^R272Q/+^ mice, and their respective wildtype littermate, we supplied plethysmography chambers with atmospheric oxygen (21% O_2_) containing 0% isoflurane. After recording the baseline activity for 45 min, we then switched to oxygen containing either 1.5% or 5% isoflurane; all mice were subjected to both 1.5% and 5% isoflurane, with sufficient rest time between exposures. To match the method of Bailey et al. [11], 1.5% isoflurane was chosen, while 5% isoflurane was chosen to rapidly induce anesthesia and potentially increase the probability of an isoflurane-induced electrographic and behavioral generalized tonic–clonic seizures. Isoflurane was delivered to mice until burst suppression was observed in the ECoG recording (e.g., Figure 2B3), indicating that isoflurane was maximally effective. Once burst suppression was observed, isoflurane delivery was halted and the animal was allowed to recover (i.e., “isoflurane recovery”). As before, video and ECoG/EMG signals were simultaneously captured. Collectively, we observed nearly identical behavioral patterns among all mice subjected to isoflurane. In total, 50% (*n* = 2/4) of *Cilk1*^+/−^, 15% (*n* = 2/13) of *Cilk1*^R272Q/+^, 50% (*n* = 1/2) of WT littermate controls for *Cilk1*^+/−^, and 50% (*n* = 1/2) of WT littermate controls for *Cilk1*^R272Q/+^ exhibited moderate head bobbing, tail extension, and occasional back and/or neck arching. These behavioral reactions were similarly consistent with opisthotonos but did not associate with any particular genotype. Importantly, the opisthotonos-like behavior did not align with any corresponding electrographic activity consistent with generalized tonic–clonic seizures in the ECoG/EMG recordings (Figure 2B–D); when present, generalized tonic–clonic seizures are readily apparent in the ECoG spectrogram. Thus, our data do not support the hypothesis that isoflurane induces generalized tonic–clonic seizures in either *Cilk1*^+/−^ or *Cilk1*^R272Q/+^ mice.

## 3. Discussion

Here, we investigated whether heterozygous *Cilk1* null mice, or mice harboring the non-functional *Cilk1* R272Q mutation, have seizure phenotypes consistent with JME. Chronic ECoG/EMG recordings revealed that *Cilk1*^+/−^ and *Cilk1*^R272Q/+^ mice do not generate spontaneous generalized tonic–clonic seizures but do occasionally produce electrographic JME-like events. However, these infrequent electrographic events occurred at the same rate as in wildtype littermates. We also tested the hypothesis that recovery from isoflurane induces tonic–clonic seizures in *Cilk1*^+/−^ and *Cilk1*^R272Q/+^ mice, as previously reported [11]. While some mice produced opisthotonos during isoflurane recovery, electrographic seizure activity was absent. In mice, opisthotonos is commonly associated with exposure to anesthesia [18]. In sum, our data do not support the hypothesis that heterozygous *Cilk1* null mice or mice heterozygous for a non-functional pathogenic *Cilk1* R272Q variant have JME. 

Juvenile myoclonic epilepsy is the most common form of the *Genetic Generalized Epilepsies* and accounts for 12% to 30% of epilepsies cared for in hospitals and clinics [23]. Imaging studies have revealed that the brains of JME patients present with altered structural connectivity [24], more cortical grey matter [25], and abnormal hippocampal structure and function [26]. Consistent with structural abnormalities, genetic screens have identified the cilia protein myoclonin1/EFHC1, a microtubule-associated protein involved in regulation of cell division, as the most frequent cause of JME [23,27,28]. Recent evidence linking *CILK1* mutations to JME underscores the potential importance of cilia in regulating the excitability of neural circuits. Bailey et al. recently identified several *CILK1* variants in JME patients and also showed that *Cilk1* haploinsufficiency in mice produces convulsions and electrographic events (i.e., spikes) associated with JME [11]; notably, only three recording days were sufficient to resolve seizures in *Cilk1* mutant mice. By contrast, our data do not support a role for either *Cilk1* haploinsufficiency or *Cilk1*^R272Q/+^ mutations as epileptogenic. While we only quantified EEG patterns from 48 continuous hours of recording, these data are representative of the ECoG/EMG activity observed over 7 days. While we did not observe a robust seizure phenotype in *Cilk1* mutant mice, we believe that concluding that *Cilk1* mutations do not form an underlying cause of JME is premature. First, differences in the mouse genetic background (i.e., BL6J versus BL6N substrains) can contribute to discrepant seizure phenotypes [29]. However, seizure phenotype is nevertheless often clear across strains for highly penetrant mutations [29]. Thus, one might expect that *CILK1* mutations strongly linked to JME, including those that impair mitosis, cell-cycle exit, and radial neuroblast migration, would also produce seizures in multiple mouse substrains. Second, not all *CILK1* mutations produce overt ciliary phenotypes. Notably, neither *Cilk1* haploinsufficiency nor *Cilk1*^R272Q/+^ mutations affect cilia growth [7,8,9]. An intriguing hypothesis is that only those *CILK1* mutations that alter cilia morphology produce JME. 

CILK1 has a conserved role in the control of cilia formation and length [3]. Knocking out both *Cilk1* alleles in mice is required to reproduce developmental phenotypes associated with altered cilia morphology and Hedgehog signaling [7,8]. In contrast, single *Cilk1* allele deletion does not produce an obvious molecular or cellular phenotype, a conclusion supported by our observation that the cortical activity of *Cilk1*^+/−^ mice was normal. 

We have recently demonstrated that overexpression of JME-associated *CILK1* variants causes exuberant cilia formation and growth in vitro [30], indicating that single residue mutations exert a dominant-negative effect on CILK1 function (wildtype CILK1 restrains cilia growth). We speculate that JME-associated phenotypes will primarily track with those *CILK1* mutations that produce overt morphological changes to cilia. Such mutations are more likely to produce deficits in neuronal maturation and migration, thereby possibly resulting in improper neural circuit formation and epilepsy. Indeed, cilia dysfunction resulting from the selective loss of ciliary GTPase Arl13b affects cilia growth and also reduces the morphological complexity of parvalbumin-positive interneurons [31], a subset of inhibitory neurons implicated in multiple forms of epilepsy [32,33,34]. Similarly, we hypothesize that *CILK1* variants that promote ciliary growth by dominant-negative mechanisms will promote similar interneuronal deficits that promote JME. Creating new rodent models harboring JME-associated *Cilk1* variants, and subsequently evaluating cilia morphology, interneuronal excitability, and seizure phenotype in these mice, will be necessary to test this hypothesis.

## 4. Materials and Methods

### 4.1. Animals

Unless otherwise stated, animals were housed at 23–25 °C under an artificial 12 h light–dark cycle with food and water provided ad libitum. *Cilk1*^R272Q/+^ and *Cilk1*^+/−^ mice [9,12] were maintained on a C57BL6/J background in the animal facilities at the University of Virginia Medical Center (Charlottesville, VA, USA). We performed experiments in mice aged P60–80. Mice of both sexes were used in all experiments—no noticeable differences were observed. 

### 4.2. Electrocorticography (ECoG)/Electromyographic (EMG) Surgery

Mouse recording devices were assembled from parts purchased at Digikey (Thief River, MN, USA). Recording devices were outfitted with insulated stainless steel wire (A-M system, Sequim, WA, USA) and stainless steel screws (Plastics One, Roanoke, VA, USA). Recording electrodes were implanted bilaterally in the cortex under 1–3% isoflurane. A reference electrode was placed in the cerebellum. A singular wire was sutured to the superficial neck muscle to obtain EMG recordings. Recording devices were secured to the skull with dental cement, and incisions were closed with sutures. Following surgery, animals received a subcutaneous injection of ketaprofen (5 mg/kg) and recovered for a minimum of 1 week before video-ECoG/EMG recording. 

### 4.3. Chronic Electrocorticography (ECoG)/Electromyographic (EMG) Recordings

Before experimentation, animals were habituated to recording cages for 48 h. ECoG/EMG signals were captured using a cable tethered to a rotating commutator (Adafruit, New York, NY, USA). To reduce movement artifact, operational amplifiers (TL2274x Texas Instruments, Dallas, TX, USA) were fixed within the recording cable. ECoG and EMG signals were filtered between 0.3 and 100 Hz and between 100 and 1000 Hz, respectively, amplified with a Model 3500 amplifier (A-M Systems), and sampled at 200 Hz with a PowerLab digitizer (ADI Instruments, Colorado Springs, CO, USA). ECoG/EMG recordings were captured using LabChart software (ADI Instruments). Video was captured using Webcam Zone Trigger software (Montreal, Quebec, CA). Chronic ECoG/EMG recordings were restarted after every 24 h for 3 days.

### 4.4. Electrocorticography (ECoG)/Electromyographic (EMG) Recording with Isoflurane

Mice were habituated to a vacuum-sealed plethysmography recording cage for 1–2 h before the day of experimentation. Recordings were performed during hours 0–12 of the 12 h light–dark cycle. On the experiment day, a 45 min baseline recording was performed with the mice exposed to room air. Subsequently, mice were exposed to two isoflurane treatments, 1.5% and 5%, interspersed with a 45 min recovery period. Isoflurane treatment stopped once burst suppression was observed in the ECoG recording. Isoflurane was rapidly expelled from the recording chamber with 100% O_2_ and room air. ECoG/EMG signals and video were captured as stated previously.

## 5. Conclusions

We are now learning that many gene mutations associated with epilepsy do not neatly fall into categories that obviously regulate neural excitability. Indeed, some have proposed that “entirely new mechanisms of epilepsy” may be identified by investigating the cell biology of epilepsy-associated genes that *do not* encode inhibitory or excitatory ion channels [35]. *CILK1* represents such a gene. We anticipate future studies to resolve how a highly conserved protein critical for fundamental cell biological processes regulates cilia function in the brain and, ultimately, interneuron maturation and neural circuit excitability.

## Data Availability

The data presented in this study are available in the article.
